# Transcriptome analysis identifies putative multi-gene signature distinguishing benign and malignant pancreatic head mass

**DOI:** 10.1186/s12967-020-02597-1

**Published:** 2020-11-07

**Authors:** Bishnupriya Chhatriya, Moumita Mukherjee, Sukanta Ray, Barsha Saha, Somdatta Lahiri, Sandip Halder, Indranil Ghosh, Sujan Khamrui, Kshaunish Das, Samsiddhi Bhattacharjee, Saroj Kant Mohapatra, Srikanta Goswami

**Affiliations:** 1 National Institute of Biomedical Genomics, P.O.: N.S.S., Kalyani, 741251 West Bengal India; 2 School of Digestive and Liver Diseases, Institute of Post Graduate Medical Education and Research, Kolkata, West Bengal India; 3 Department of Surgery, R G Kar Medical College and Hospital, Kolkata, West Bengal India; 4 Chittaranjan National Cancer Institute, Kolkata, West Bengal India

**Keywords:** Pancreatic head mass, Transcriptome, Signature, Biomarker potential

## Abstract

**Background:**

Most often, the patients with pancreatic diseases are presented with a mass in pancreatic head region and existing methods of diagnosis fail to confirm whether the head mass is malignant or benign. As subsequent management of the disease hugely depends on the correct diagnosis, we wanted to explore possible biomarkers which could distinguish benign and malignant pancreatic head masses.

**Methods:**

In order to address that gap, we performed a case–control study to identify genome-wide differentially expressed coding and noncoding genes between pancreatic tissues collected from benign and malignant head masses. These genes were next shortlisted using stringent criteria followed by selection of top malignancy specific genes. They subsequently got validated by quantitative RT-PCR and also in other patient cohorts. Survival analysis and ROC analysis were also performed.

**Results:**

We identified 55 coding and 13 noncoding genes specific for malignant pancreatic head masses. Further shortlisting and validation, however, resulted in 5 coding genes as part of malignancy specific multi-gene signature, which was validated in three independent patient cohorts of 145 normal and 153 PDAC patients. We also found that overexpression of these genes resulted in survival disadvantage in the patients and ROC analysis identified that combination of 5 coding genes had the AUROC of 0.94, making them potential biomarker.

**Conclusions:**

Our study identified a multi-gene signature comprising of 5 coding genes (*CDCA7*, *DLGAP5*, *FOXM1*, *TPX2* and *OSBPL3*) to distinguish malignant head masses from benign ones.

## Background

Pancreatic cancer is one of the most aggressive forms of cancers, with 5-year survival as low as 7 per cent. Chronic pancreatitis (CP) is considered as a major risk factor for pancreatic cancer. In about 30–75% of CP cases, a benign inflammatory mass is formed in pancreatic head region which is very much similar to malignant pancreatic head mass which occurs in about 65–70% of PC [1]. Jaundice, gastric outlet obstruction, weight loss, back-ache are symptoms common to both. Diagnosis is difficult even during the surgery as features like hard mass, vascular invasion are present in both the cases. Distinguishing benign and malignant head mass based on their clinical and imaging features is very challenging but necessary as they have very different treatment and management strategies. Misdiagnosis of benign head mass as malignant head mass will result in unnecessary surgical treatment and misdiagnosis of malignant head mass as benign head mass could result in unnecessary delay in required treatment. In doubtful situations radical approach is used and pancreaticoduodenectomy is performed. The situation further worsens in regions where tropical calcific pancreatitis (TCP) is more common. Pancreaticoduodenectomy in those patients is associated with very high post-operative morbidity as the patients are nutritionally deficient due to exocrine and endocrine insufficiency [2, 3]. So, the need of distinction between benign and malignant is even more pronounced in tropical country like India. It has been shown that integration of dynamic contrast-enhanced CT scan, MRI and ^18^F-FDG-PET/CT imaging methods could be used for differential diagnosis of benign and malignant head mass, but evidences are still not strong enough [4]. Platelet-Lymphocyte ratio (PLR) along with CA19-9 has been shown to address the issue to some extent but they have their own limitations [5–7].

Hence, there is an urgent need for the identification of some other parameter or method which could distinguish between the two types of head masses confirmatively. There are also studies looking into the proteome profile of pancreatic cancer and pancreatitis but with not much success [8, 9]. Analysis of transcriptome has also been used to distinguish benign and malignant lesions in other cancers [10, 11], but such studies directly addressing issues with benign and malignant pancreatic head masses are lacking. The importance of transcriptome analysis is that the investigation of identified DEGs not only helps us to derive and validate potential signatures specific for diagnosis of a disease condition but also help to understand the biology as well.

In this study, we performed gene expression analysis between benign and malignant pancreatic head masses and identified differentially expressed mRNAs and noncoding RNAs. In the next step, a small set of markers was carefully shortlisted and validated by qRT-PCR. Additionally, their expression was checked in TCGA pancreatic cancer datasets and other publicly available datasets and our findings were consistently replicated. Finally we performed ROC analysis and were able to propose a 5 gene signature that can effectively distinguish malignant pancreatic head masses from benign.

## Methods

### Sample collection

Tissue samples were collected from patients undergoing surgery at IPGME&R, Kolkata, RG Kar Medical College and Hospital, Kolkata and Chittaranjan National Cancer Institute, Kolkata for benign pancreatic disease condition i.e. chronic pancreatitis as well as for malignant pancreatic disease condition i.e. pancreatic cancer. Additionally, adjacent normal pancreatic tissue samples were also collected and all tissue samples were stored in RNA-Later. All the tissues were collected while performing the surgery after careful investigation of the head mass. Histopathological examination by expert pathologists confirmed whether they were malignant or benign in nature. For the present discovery set investigation, 9 normal, 6 CP and 11 PC samples were selected and 9 CP and 9 PC samples were selected for validation. Relevant patient information has been given in Additional file 1: Table S1.

### RNA extraction

About 20 mg of tissue sections were taken for each samples and total RNA was extracted according to the instructions mentioned in the manual of All-Prep DNA/ RNA/ miRNA isolation kit from Qiagen (catalog number: 80224). Quantification was done using multi-channel spectrophotometer (Model number: ND 8000, Thermo Fisher Scientific). Quality of RNA was checked by denaturing agarose gel electrophoresis for characteristic RNA bands.

### Microarray

Gene expression profiling was done by microarray using Affymetrix human transcriptome array 2.0 (HTA 2.0) platform, which consists of probes for both coding and non-coding genes. cDNA was prepared from ~ 10 µg of RNA, biotinylated according to standard Affymetrix protocol and then hybridized onto Affymetrix HTA 2.0 Arrays for overnight in the hybridization oven and then the array chips were washed and stained in the Affymetrix Fluidics Station 450. GeneChips were scanned using the Affymetrix GeneChip® Scanner 3000 7G and raw files were obtained as CEL files.

### Data acquisition and pre-processing

Raw data were obtained as CEL files which were further pre-processed before calculating differential expression. The raw data were first read into R as an affybatch object and an expression set is created as an expression set object. An expression set object is created from affybatch object and then pre-processed for background correction, normalization, probe summarization. Background correction and probe summarization was done by using “Oligo” package of R Bioconductor [12]. Normalization was done by ‘Invariant Set Method’ using “affyPLM” package of R [13]. In this method of normalization, a set of genes whose expression is consistent in all the samples were identified and based on those expression values, the expression values of other genes were adjusted in each sample. The raw and processed data have been submitted to GEO (GSE143754).

### Quality control analysis

The quality of array was checked using “array-Quality-Metrics” package in R [14]. It has four parameters for checking the quality, as follows: (a) between array comparisons: The difference between the arrays was checked using principal component analysis and distance between individual arrays (Additional file 2: Figure S1). (b) Array intensity distributions: These were checked by boxplots and density plots (Additional file 3: Figure S2). (c) Variance mean dependence: It was checked by plotting a graph between standard deviations in y-axis versus rank of mean of intensities in x-axis (Additional file 4: Figure S3). (d) Individual array quality: They were checked by plotting MA plots (Additional file 5: Figure S4).

### Differential expression

The differential expression between study groups was calculated using the “limma” package of Bioconductor after removing non-specific probes [15]. Model matrix was designed based on the study groups. The expression was fitted onto a linear model and then contrasts were generated. Empirical Bayes moderation of the standard errors was performed for computing moderated t-statistics and detection of differential expression. The p-values obtained were further corrected for multiple testing by Benjamini–Hoechberg method [16]. Adjusted p-value of 0.05 and absolute fold change of 2 < FC > -2 was then imposed to identify the differentially expressed genes. DEGs from different groups were compared using Venny 2.1 [17].

### Annotation of genes

Genes were annotated to KEGG and Reactome pathways, OMIM and GAD diseases, GO terms and UniProt keywords using DAVID functional annotation tool [18].

### Validation of genes

**Fig. 1:**
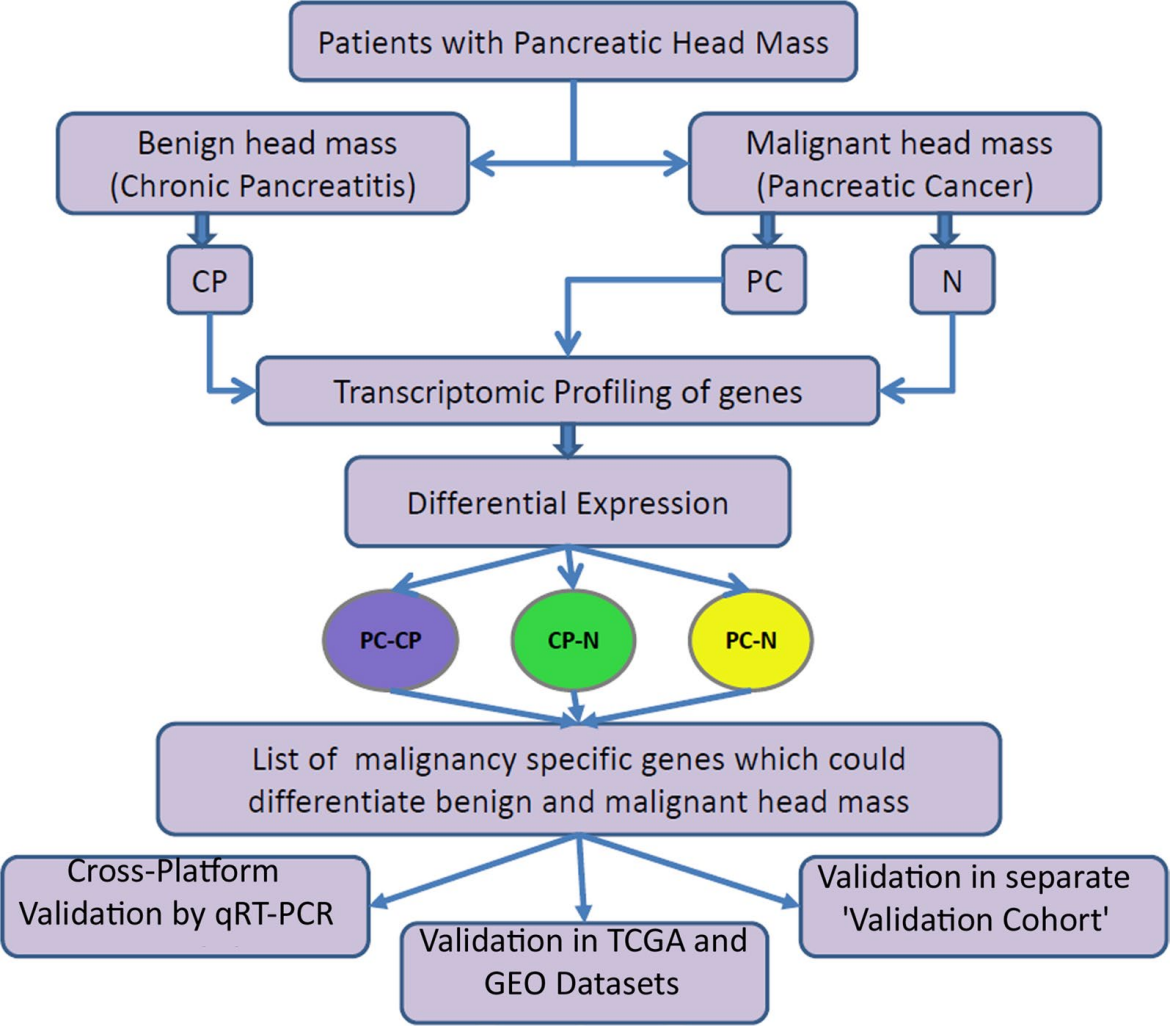
Schematic flowchart showing the study design followed in this study

The expression of genes was validated by quantitative RT-PCR using PMM1 as housekeeping gene (expression level of GAPDH was found to be altered in our results). The primer sequences are given in Additional file 6: Table S2. The experiment was carried out in Applied Biosystem Step One Plus Instrument using Luna Universal One-Step RT-qPCR Kit (Catalog number: E3005X) from New England Biolabs, USA; following manufacturer’s instructions. Unpaired t-test was performed for statistical significance. Gene expression was further validated in pancreatic adenocarcinoma (PAAD) dataset of TCGA data using the web-tool GEPIA [19]. Moreover, a meta-analysis was done to identify differential expression status of the selected genes. The normalized data from GSE62452, GSE15471 and GSE28735 were combined to form a single expression set and batch effects were corrected using comBat function of “sva” package in R [20]. Differential expression was calculated using “limma” package of Bioconductor [15]. Log2 transformed data was used to calculate fold change or relative expression between benign and malignant groups. The characteristics of each dataset are described in Additional file 7: Table S3. Lastly, we have used a fresh set of 9 benign and 9 malignant pancreatic head mass tissues and validated the gene signature in them by quantitative RT-PCR. The overall plan has been shown in Fig. 1.

### Survival analysis

Survival analyses were done in PAAD dataset of TCGA data using the web-tool GEPIA [19]. Kaplan–Meier survival curves were plotted for the 178 pancreatic cancer samples were divided into two groups based on high and low expression of the genes respectively. Then log rank test was done to compare both the curves. Hazard ratio was also calculated to find out the difference between high expression group and low expression group. A p-value of less than 0.05 was considered significant for both log rank test and hazard ratio.

### ROC Analysis

GSE62452, GSE28735 and GSE15471 were used as validation cohort for ROC analysis in multiple levels.

- Level 1: Here, ROC curve was generated and sensitivity, specificity and AUROC were calculated for each gene in each of the three datasets. This was done using “ROCR” package in R [21].
- Level 2: A single ROC curve for each gene in the merged dataset was generated as in ‘Level 1’.
- Level 3: In order to evaluate the combined biomarker potential of the genes, a ROC curve was generated after combining the genes by linear modeling in a cross validation approach. The detail steps are as follows:

Correlation of genes in merged datasets: Gene expression correlation was checked for the five selected coding genes by using “Hmisc” and “corrplot” package of ‘R’ [22].

Data partitioning: Then the combined data was partitioned into ‘training set’ with 70% of the samples and ‘test set’ with remaining 30% of the samples. Thus, ‘training set’ was created with 107 PC samples and 104 normal samples and ‘test set’ were created with 46 PC samples and 41 normal samples. Data partitioning was done using “caret” package of R [23].

LASSO regression model: After data partitioning, a 10-fold cross validation approach was taken to generate a Lasso regression model from the training set using all the 5 genes and their interaction terms and predictions were made on the test set. This was done using the R package “glmnet” [24]. Then sensitivity, specificity and AUROC was calculated from the predictions using “ROCR” package in R [21]. The overall plan has been shown in details in Additional file 8: Figure S5.

## Results:

Our primary objective was to distinguish benign and malignant pancreatic head masses. We chose to explore key transcriptomic alterations and focused on both coding and long noncoding RNA expression changes. It is established fact that SNPs modulate gene expression due to their variation in different populations [25] and we don’t have much information regarding genetic alteration of PDAC patients in India. Therefore, instead of combining our results with other published reports and do a meta-analysis, we decided to validate the findings of our patients in TCGA data and other expression datasets to assess their importance. Subsequent to identification of differentially expressed genes and adequate statistical testing, we selected malignancy specific genes which do not alter between normal and CP but changes in PC. Resulting 55 coding and 13 noncoding genes were further validated by qRT-PCR, in TCGA dataset and three other datasets from GEO. Survival analysis was performed and their biomarker potential was also investigated.

## Identification of differentially expressed genes

To distinguish benign and malignant pancreatic head masses, we chose to explore their transcriptomic profiles via microarray analysis. Total RNA was isolated from surgically resected head mass tissues and gene expression patterns of both coding and noncoding RNAs were analysed in three study groups of 9 normal (N), 6 chronic pancreatitis (CP) and 11 pancreatic ductal adenocarcinoma (PC) patients. After comparison between themselves, three differential gene expression patterns were obtained as described below:

- CP vs N: In this comparison 7 upregulated and 181 downregulated coding genes were obtained. Along with that we also obtained 148 upregulated and 11 downregulated non-coding genes.
- PC vs N: Similarly we obtained 367 upregulated and 249 downregulated coding genes, along with 40 upregulated and 75 non-coding genes in this comparison.
- PC vs CP: We got 894 upregulated and 124 downregulated coding genes along with 47 upregulated and 475 downregulated non-coding genes in this comparison.in this comparison.

While heat maps demonstrate the expression status of different genes between two groups, volcano plots show the fold change along with their significance. Additional file 9: Figure S6; panel A, B and C shows volcano plots corresponding to the coding genes compared between three groups while Additional file 9: Figure S6; panel D, E and F shows the respective heat maps. Similarly, Additional file 10: Figure S7; panel A, B and C and Additional file 10: Figure S7; panel D, E and F shows the volcano plots and heat maps for noncoding gene expression comparisons. The lists of differentially expressed genes (both coding and noncoding) resulting from these three comparisons are given as Additional file 11: Table S4, Additional file 12: Table S5, Additional file 13: Table S6, Additional file 14: Table S7, Additional file 15: Table S8 and Additional file 16: Table S9.

## Identification of malignancy specific gene signature

**Fig. 2:**
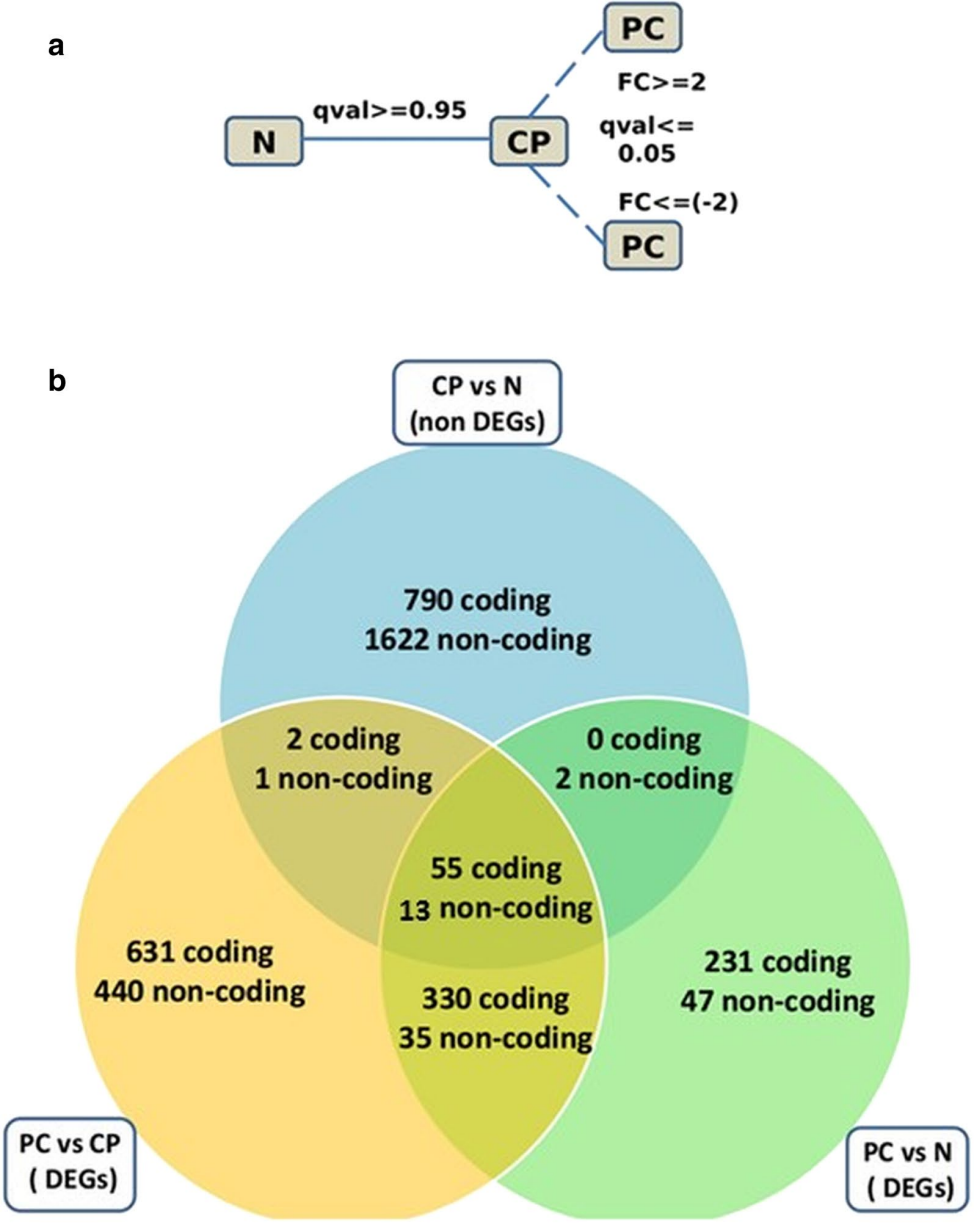
Identification of malignancy specific genes: **a** The criteria used for identification and **b** the number if genes identified following this criteria for each of the comparisons

For a gene to be specific for malignancy, its expression should be unaltered in ‘normal’ (N) and ‘chronic pancreatitis’ (CP) samples as both are benign condition and deregulated in ‘pancreatic cancer’ (PC) i.e. in malignant condition. In other words, a gene specific for malignancy should not be differentially regulated in CP vs. N comparison but significantly deregulated in PC vs. N and PC vs. CP comparisons. The q-value and fold-change criteria used for this selection is shown in Fig. 2, panel a. Here q-value refers to fdr (false discovery rate) corrected p-value. The criteria was chosen such that top genes specific for malignancy are selected. The first criterion was to select the gene whose expression was not altered in CP as compared to N, both of them being benign conditions. So a q-value ≥ 0.95 was used to select such genes. The second criterion was to select the gene whose expression is altered in PC as compared to CP. Using both the criteria we identified 55 coding and 13 non-coding genes as shown in Fig. 2, panel b. The complete list of the malignancy specific genes is shown in Table 1. Further short-listing of both the coding and noncoding genes were made based on their consistent expression in all the cases as compared to controls and also based on previous reports of the same in solid tumours, as shown in Additional file 17: Figure S8.

**Table 1:** List of the malignancy specific genes identified in this study

Coding genes up-regulated in malignancy
*NQO1, SERPINB5, CLRN3, ACSL5, CENPE, LGR5, SLC6A14, TPX2, SLC5A1, SMIM24, NDC80, FAM111B, ASPM, FAM72D, CDCA7, VSIG1, DLGAP5, GUCY2C, B2M, CKAP2, C17orf78, CYP2C18, POSTN, SLC44A4, CCNB2, OTC, LRRC66, CCL24, GDA, OSBPL3, SEMA3C, CDC6, TMC7, KIF4A, MYOA1, FOXM1, MYOF, GPA33, ABCG2, IL2RG, LAMA3, PLEKHA2, C6orf47, RAD51AP1, ANO1, ADAM28, NAPEPLD, HPGD, KIAA1551, GBP1, MED27*
Coding Genes Down-regulated in malignancy:
*CPA1, REG1B, C7, SPP1*
Non-coding Genes Up-regulated in malignancy:
*SAMD12-AS1, LINC00263, LINC00294, RCN1P2, MCTS2P, LINC01133*
Non-coding Genes Down-regulated in malignancy:
*SNORD116-1, SNORD116-3, SNORD116-5, SNORD116-29, SNORD115-15, LOC100506281, SNORA76C*

## Annotation of genes

Once we have the differentially expressed genes identified, it is very important to know what is their functional importance and what are the major biological pathways they alter or what are the diseases they are associated with. Malignancy specific genes annotated to KEGG and Reactome pathways and GAD diseases, GO terms and UniProt keywords. We found that these genes contributed to various pathways including Cell cycle, pancreatic secretion, cytokine-cytokine receptor interaction, chemokine signaling, metabolic pathways and signaling pathways like p53 signaling pathway, *PI3K-Akt* signaling pathway, FoxO signaling pathway among the important ones. Furthermore, many of the genes could also be annotated to diseases like cancer, immunity-related diseases, aging and metabolic diseases according to Genetic Association Database (GAD) [26]. The detailed results could be found in Additional file 18: Table S10.

## Evaluation of the gene expression status in Global dataset of PDAC

**Fig. 3:**
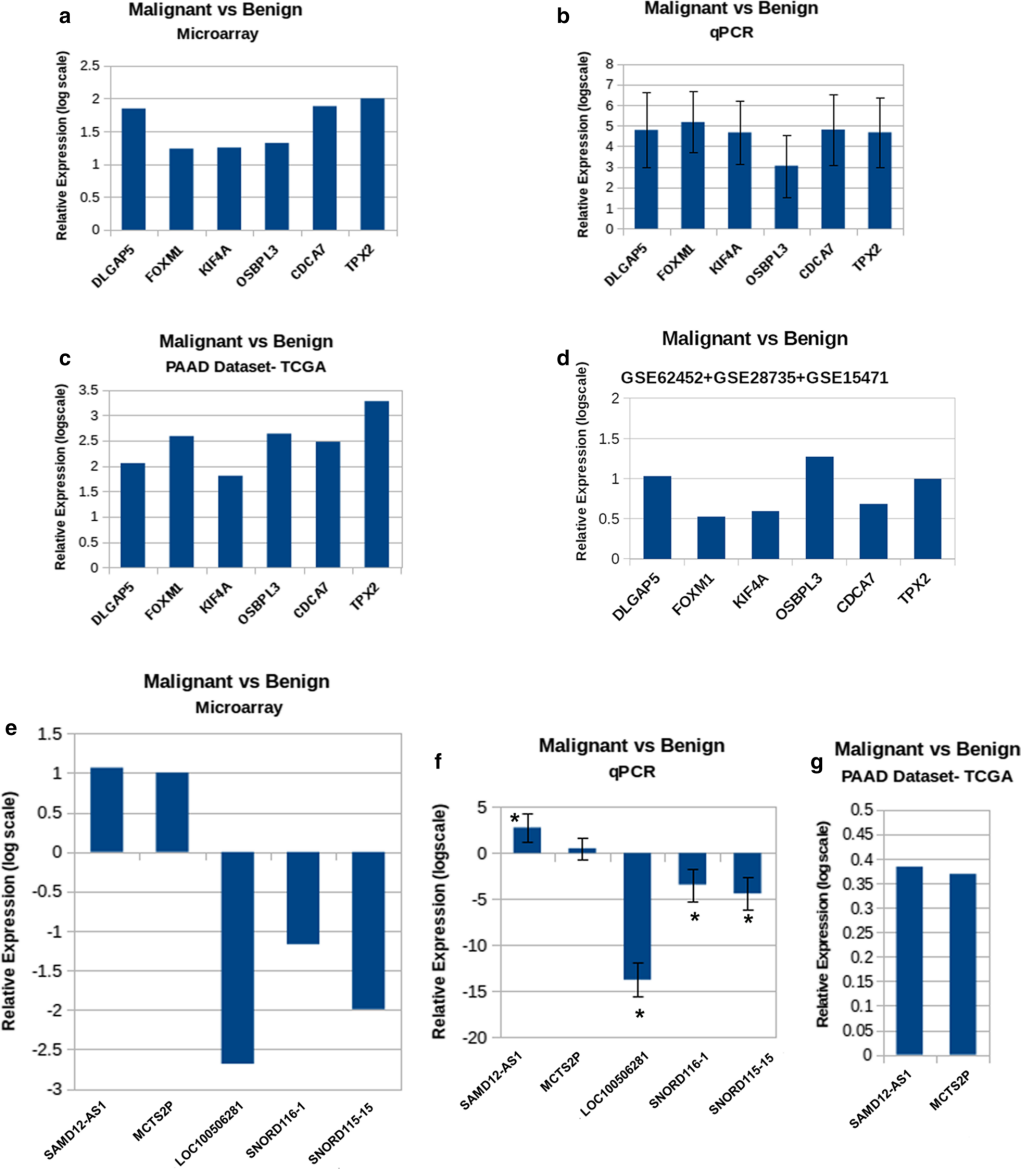
Evaluation of gene expression in global datasets: Level of gene expression for selected coding genes in **a** microarray, **b** qRT-PCR, **c** PAAD Dataset of TCGA and **d** GEO datasets. Level of gene expression for selected non-coding genes in **e** microarray, **f** qRT-PCR and **g** PAAD Dataset of TCGA. Error bars in (**b**) and (**f**) represents standard deviation. All the expression values in (**a**–**d**) and (**f**) are statistically significant. Only the expression values marked with (*) are significant in (**e**)

We selected 7 top hits from both coding and noncoding malignancy specific genes (Additional file 17: Figure S8) and initially performed the cross-platform evaluation by testing their expression by quantitative RT-PCR. We could confirm expression of 6 coding genes and 5 noncoding genes as shown in Fig. 3. The qRT-PCR results corroborated with the microarray results and confirmed significant deregulation of those genes as seen in high-throughput studies (panel b). Furthermore, we also wanted to test the nature of expression of these genes in TCGA data. We followed the GEPIA web-tool and found all of our selected coding genes were also significantly altered in same direction in the TCGA pancreatic cancer samples (panel c). They might not be top hits in that population, but their similar deregulation supports that what we have found must be important in disease context. However, we couldn’t validate three of the noncoding genes in TCGA dataset as it didn’t have expression information for them. They got validated only by qRT-PCR (panel g). Additionally, the expression status of these 6 coding genes was also checked in the combined data generated from GSE62452, GSE28735 and GSE15471 (shown in Additional file 7: Table S3). All the 6 genes were found to be significant with adjusted p-value < 0.05 (Fig. 3d).

## Survival Analysis

**Fig. 4:**
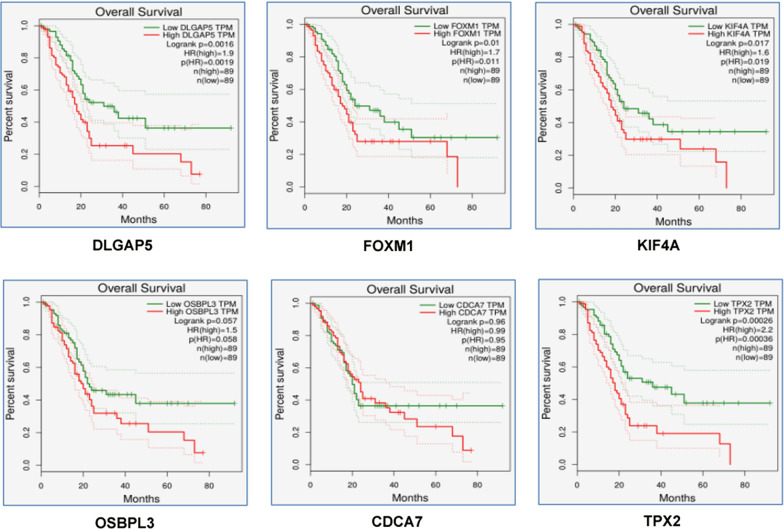
Survival analysis: The survival plots with Kaplan–Meier curves for the selected coding genes as mentioned

It is apparent that a gene important for oncogenesis, especially, if involved in metastasis, will have its direct influence on patient survival. We wanted to assess whether our malignancy specific coding and noncoding gene signatures are also responsible for poor-prognosis. Survival analysis was done for them in 178 samples of PAAD dataset from TCGA using GEPIA to identify the total survival estimates. Statistically significant difference was observed in Kaplan–Meier plots between higher expression group and lower expression group for *DLGAP5, FOXM1, KIF4A* and *TPX2*, where higher expression of these genes shows poor prognosis and overall survival disadvantage as shown in Fig. 4. We have also performed the disease-free survival analysis and the results are similar (data not shown). In other words, these genes seem not only to be important for distinguishing pancreatic malignancy from benign conditions but also important for prognosis of the disease. However, we could only perform the analysis using two of the selected noncoding genes (*SAMD12-AS1* and *MCTS2P*) and both of them didn’t show any significant survival advantage. As mentioned before, because of the unavailability of expression information in TCGA dataset, we couldn’t perform the survival analysis for *LOC100506281*, *SNORD116-1* and *SNORD115-15*.

## Validation of our results in a new Pancreatic Head Mass Cohort

**Fig. 5:**
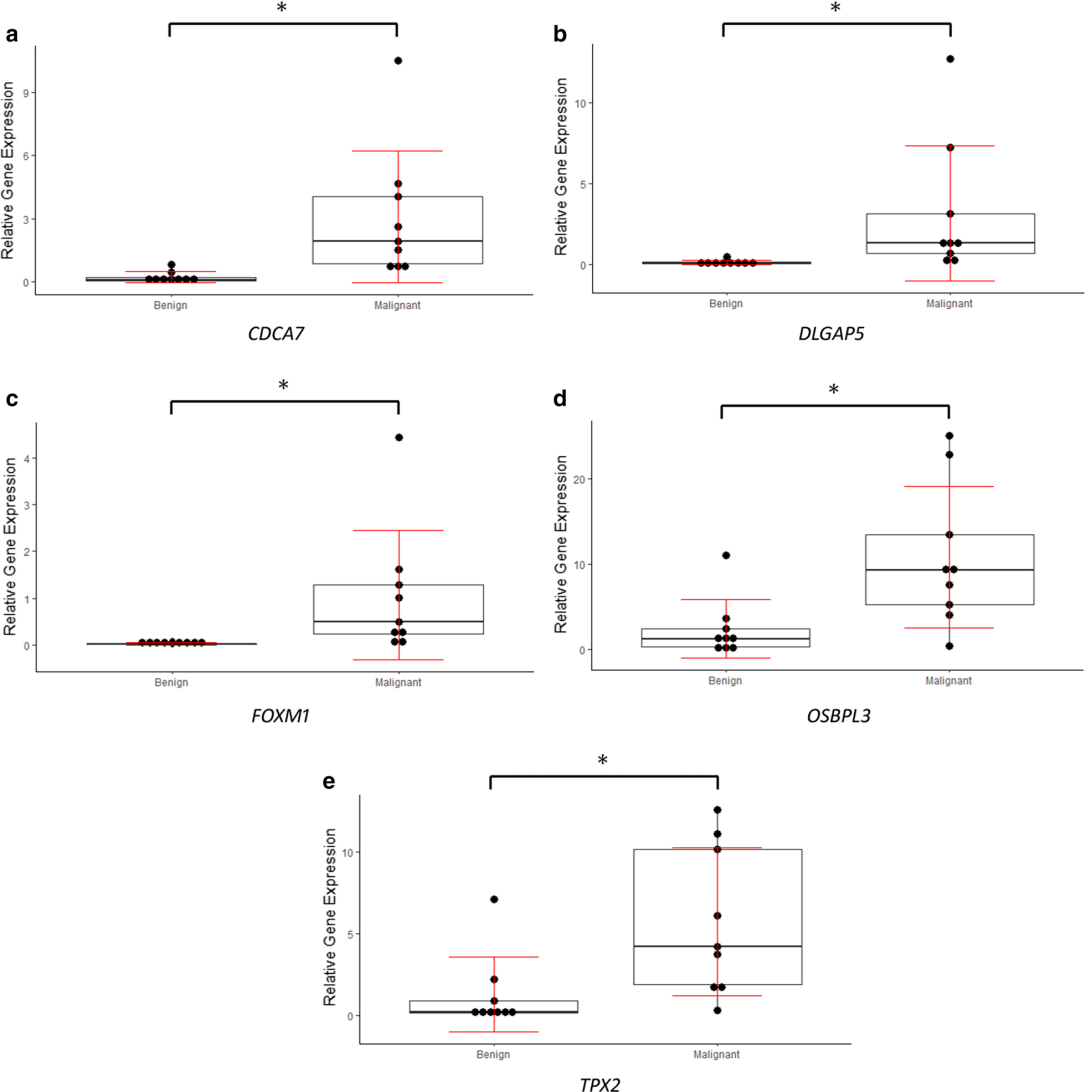
Validation of gene expression in a separate cohort: **a**–**e** the expression of the respective genes in 9 benign and 9 malignant head mass tissue samples, as measured by qRT-PCR. 2^-∆Ct values are plotted against the ‘Y’ axis, denoted by ‘relative gene expression’, where the individual dot represents the normalized expression value (with respect to house-keeping gene *PMM1*) of that particular gene in that particular sample. The difference in expression values marked with (*) are statistically significant (p-value < 0.05/ unpaired t-test)

Initial validation of the six coding gene signature using global gene expression datasets like TCGA and GEO confirmed that these genes are significantly deregulated in pancreatic tumour tissues from patients all over the world. However, it is known that around 65–70% of all the malignancy in pancreas is anatomically located in ‘pancreas head’ [27]. Hence, validation of our multi-gene signature in global pancreatic cancer datasets might not accurately reflect their expression status in malignant pancreatic head masses. Therefore, to have a more conclusive picture, we further took a fresh set of 9 benign and 9 malignant pancreatic head mass tissue samples and checked the expression of all these genes in them by quantitative RT-PCR. We found that five among the six genes (*DLGAP5, CDCA7, FOXM1, TPX2*, and *OSBPL3*) were still significantly upregulated in malignant pancreatic head masses (Fig. 5a–e). *KIF4A* couldn’t survive the validation analysis. The finding clearly confirms the candidature of those five genes to be tested for their biomarker potential for detection of malignant pancreatic head masses.

## ROC Analysis

**Fig. 6:**
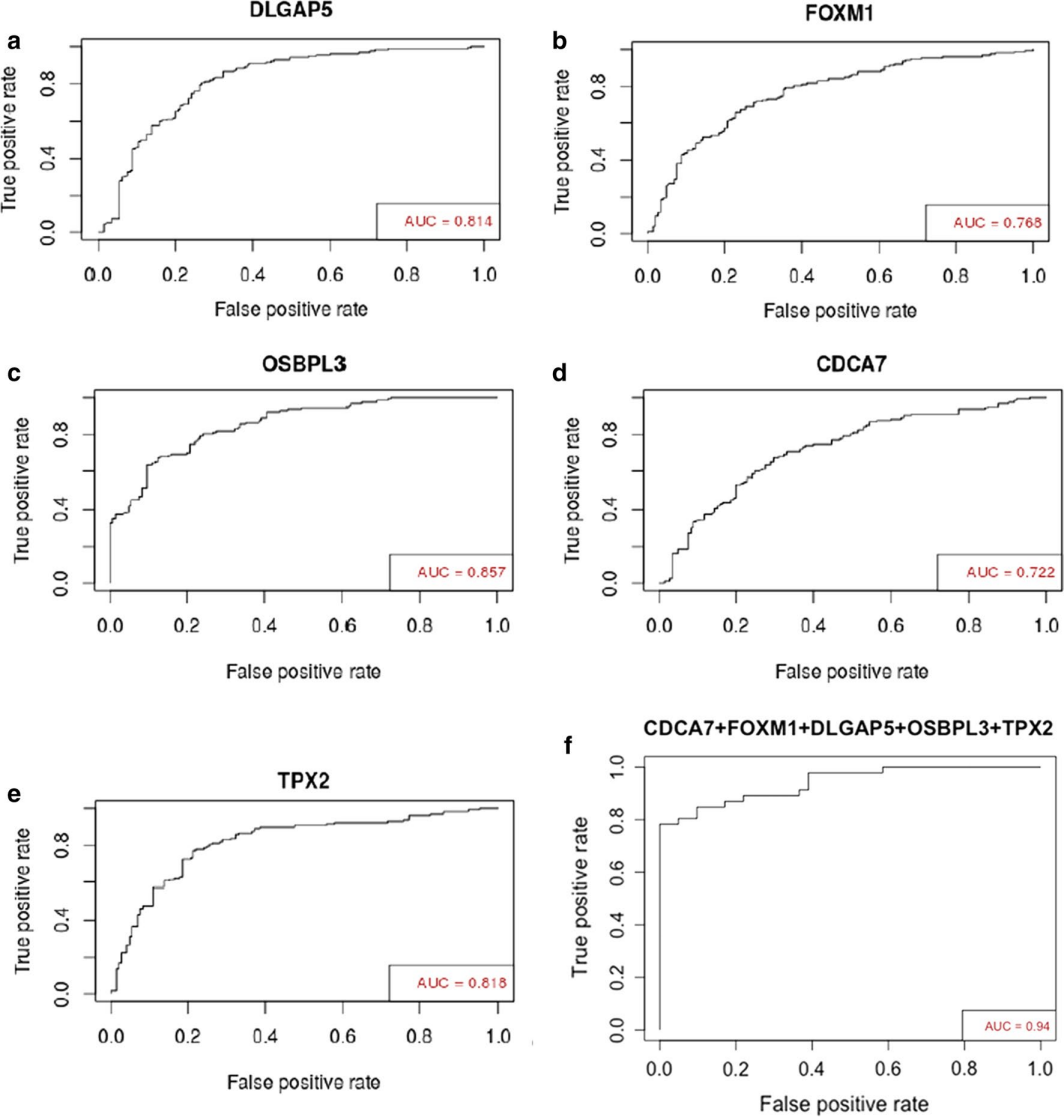
ROC Analyses: **a**–**e** ROC plots for the selected coding genes individually and **f** ROC plot of the combined genes

Finally, we planned to perform the ROC analysis to assess the biomarker potential of the validated coding gene signatures for pancreatic malignancy using datasets GSE62452, GSE28735 and GSE15471. As described in Additional file 8: Figure S5, we followed rigorous analysis methods. Firstly, analysis was done individually for each gene in each of the three dataset (Table 2). Secondly, we combined these three datasets and analyzed the AUROC of each of the genes in the combined or merged dataset and found that values for the all the genes were quite impressive. Next, in order to find out the combinatorial effect of the genes, we first looked at correlation of their respective expression pattern in the merged dataset and found that the genes had varied degree of correlations among themselves. Expression of *DLGAP5, FOXM1* and *TPX2* emerged to be as most correlated (Additional file 19: Figure S9). In order to improve the results further, all the five genes were combined and the analysis was repeated in the merged dataset. The diagnostic potential of multi-gene biomarker was found to be better than individual gene biomarkers with AUROC of 0.94, 84.78% sensitivity and 90.24% specificity. Detailed result is given in Table 2 and Fig. 6 shows the AUROC plots for all the genes in the merged dataset.

**Table 2:** ROC parameters

Gene	Parameters	GSE15471	GSE28735	GSE62452	Merged Dataset
*CDCA7*	Sensitivity	0.769	0.689	0.812	0.673
	Specificity	0.667	0.822	0.639	0.703
	AUROC	0.736	0.79	0.747	0.722
*DLGAP5*	Sensitivity	0.769	0.889	0.87	0.804
	Specificity	0.692	0.756	0.721	0.731
	AUROC	0.779	0.842	0.83	0.814
*FOXM1*	Sensitivity	0.821	0.778	0.681	0.712
	Specificity	0.641	0.756	0.738	0.724
	AUROC	0.721	0.817	0.764	0.768
*OSBPL3*	Sensitivity	0.769	0.844	0.783	0.804
	Specificity	0.744	0.778	0.803	0.759
	AUROC	0.87	0.856	0.852	0.857
*TPX2*	Sensitivity	0.718	0.844	0.826	0.771
	Specificity	0.744	0.844	0.754	0.786
	AUROC	0.743	0.850	0.837	0.818
*CDCA7* + *DLGAP5* + *FOXM1* + *TPX2* + *OSBPL3*	Sensitivity	–	–	–	0.847
	Specificity	–	–	–	0.902
	AUROC	–	–	–	0.942

This table shows the ROC parameters for the selected genes individually in each datasets, merged dataset as well as all genes combined in merged dataset

## Discussion:

In majority of the cases the inflammatory mass resulting from chronic pancreatitis occurs in the head region of the pancreas. On the other hand, malignant pancreatic head masses are also predominant in the pancreatic head. Hence, it is a diagnostic dilemma for the clinicians when a patient comes to the clinic with a pancreatic head mass, whether the mass is benign or malignant. We wanted to identify potential biomarkers capable of distinguishing these two types of masses and set out to explore the transcriptome profile between them. We didn’t restrict our investigation to coding genes but focused on both coding and noncoding genes together and our differential gene expression analysis identified relevant alterations for each of the three pairs of groups compared (Additional file 9: Figure S6 and Additional file 10: Figure S7). Further analysis of malignancy specific gene expression pattern identified a set of 55 coding and 13 noncoding genes differentially expressed in malignant head masses as compared to benign ones (Table 1). As described in ‘Results' section corresponding to Fig. 2, we focused our analysis to identify genes which are unaltered in normal pancreas and benign head mass both, but significantly changed in malignant conditions. This list included both up and down regulated genes and could be a good starting point to explore their functions and importance as potential diagnostic marker. We further explored the functional annotations of these deregulated genes and found their involvement in major biological pathways as well as could connect their expression alterations to specific diseases like cancer among others. The finding, as detailed in Additional file 18: Table S10, supports the importance of the DEGs we identified in pancreatic carcinogenesis.

Next, we rearranged the list to find out the genes which are mostly altered in all of our samples and concentrated on the top hits among them. Thus, 7 coding and 7 noncoding genes were selected for subsequent validation. We performed cross-platform validations by qRT-PCR and could validate 6 coding and 5 noncoding genes and subsequently checking their expression in TCGA and GEO datasets confirm similar finding in patients belonging to other populations.

Incidentally, all of these six genes were found to be upregulated and was reported as promoters of tumourigenesis. However, there are no reports of *CDCA7* and *KIF4A* of their involvement in PDAC. *CDCA7* is cell division cycle associated protein-7 which is a *c-MYC* responsive gene and its role in *c-MYC* dependent tumourigenesis has been established by several studies [28, 29], while *KIF4A* is a member of kinesis family known to be a predictor and prognostic marker for hepatocellular carcinoma, oral and colorectal cancer [30–32]. We report here for the first time involvement of *CDCA7* and *KIF4A* in PDAC. On the other hand, there are multiple reports showing *TPX2* and *FOXM1* being involved in development and progression of PDAC [33–36]. *TPX2* is microtubule nucleation factor while *FOXM1* is a member of Forkhead box transcription activator proteins involved in cell proliferation. *DLGAP5* is *DLG* associated protein 5, thought to play multiple roles in carcinogenesis and has been established as a promising early detection biomarker for lung adenocarcinoma and bladder cancer [37–39]. Interestingly, one bioinformatics study has also identified *DLGAP5* as a progression biomarker for PDAC, which, in turn, supports our finding [33]. The last one is *OSBPL3*, oxysterol binding protein like-3, involved in cell adhesion and organization of actin cytoskeleton. The important fact about this gene is that it has emerged as one of the novel predictive biomarker for PDAC in an integrative gene expression profiling analysis, further endorsing our results [40]. In case of noncoding genes, we didn’t find much information from published literature. Among the two upregulated noncoding genes, the most important is *SAMD12-AS1*, known to promote malignant progression in glioma and high-risk neuroblastoma [41, 42]. We are the first to report the possible involvement of this long noncoding RNA in pancreatic malignancy. The other upregulated noncoding gene is *MCTS2P*, which is a pseudogene for *MCTS1*, a critical cell cycle regulator. No available information is there regarding contribution of this long noncoding RNA in cancer. With respect to the down regulated noncoding genes, we report *LOC100506281*, *SNORD116-1* and *SNORD115-15* found to be down regulated for the first time in PDAC. Interestingly enough, *LOC100506281,* another long noncoding RNA, has not been associated with any pancreatic disease before. However, it has been reported as hugely overexpressed in normal pancreas [43]. Therefore, our observation of its down regulation in PDAC could be necessary for tumourigenesis and would really be worth exploring for further details. Similarly, snoRNAs *SNORD115* and *SNORD116* are considered to be orphan C/D box snoRNAs and reported to alter expression of multiple genes [44]. Members of these families of RNAs have been found to be involved in tumourigenesis and loss of these gene clusters has also been linked with other diseases [45, 46]. *SNORD116* gene cluster has also been found to be important for development of pancreas. Thus, detailed exploration of published reports on our top selected genes shows that we have found some new players both in terms of coding and noncoding genes, significantly altered in our patients with malignant head mass. This further supports our initial hypothesis that there could be population specific differences in gene expression. Furthermore, the higher expression of our upregulated genes demonstrated poor survival of the patients (Fig. 4) and *DLGAP5, FOXM1, KIF4A* and *TPX2* emerged having significant survival disadvantages when overexpressed, indicating their possible involvement in poor-prognosis. However, establishment of this fact needs further investigation. Unfortunately, we couldn’t perform similar analysis for the noncoding genes as the expression information of those genes were not available in that database.

We have increased the stringency of selection criteria of the genes by another level considering the fact that validation in TCGA or GEO datasets of pancreatic cancer might not exactly reflect the true scenario as results from malignant head masses constitute a fraction in them. Hence we further validated expression of those 6 coding genes in additional 9 benign and 9 malignant pancreatic head mass tissue samples and *DLGAP5, FOXM1, CDCA7, TPX2* and *OSBPL3* showed similar expression pattern. Finally, we performed the ROC analysis at multiple levels (Fig. 6) using the individual genes where all of them had impressive AUROC values (Table 2). The obvious approach at this point was to evaluate whether the combined multi-gene signature could perform better and we found the plot to have AUROC of 0.942, clearly having diagnostic edge over any of the single genes. In this context, it will be important to mention that CA19-9 has been used clinically for the diagnosis of Pancreatic Cancer. In various systematic reviews, it has been found that the sensitivity of CA19-9 is in the range of 78–81% and specificity is in the range of 80–85% [47–49]. The combined sensitivity and specificity we are getting is better than CA19-9 alone. However, we couldn’t evaluate CA19-9 in combination with the five-gene signature as none of these datasets had relevant CA19-9 information for the patients.

## Conclusions:

Therefore, considering the importance of diagnostic dilemma related to the nature of pancreatic head mass, here we have first identified a set of differentially expressed coding and noncoding genes between benign and malignant pancreatic head masses. Next, we validated the top deregulated genes by qRT-PCR in separate validation cohort and also in TCGA and GEO datasets and reported a multi-gene signature of 5 coding genes (*CDCA7, DLGAP5, FOXM1, TPX2* and *OSBPL3*) capable of acting as potential biomarker to distinguish malignant pancreatic head masses from benign ones.

## Supplementary information

**Additional file 1: Table S1.** Patient characteristics: This table shows the patients characteristics for the samples used in this study

**Additional file 2: Figure S1.** Array quality metrics-Between array comparisons: This figure shows between-array comparisons. (A) shows the distance between arrays and (B) shows Principal Component analysis (PCA).

**Additional file 3: Figure S2.** Array quality metrics-Array intensity distributions: This figure shows the array intensities in (A) boxplots and (B) density plots

**Additional file 4: Figure S3.** Array quality metrics-Variance Mean dependence: This figure shows the Variance Mean dependence of the arrays, where the red line connect the medians of each probe in the arrays.

**Additional file 5: Figure S4.** Array quality metrics-Individual array quality: This figure shows the MA plots where, M = log 2 (I_1_)- log 2 (I_2_) and A = 1/2 (log 2 (I_1_) + log 2 (I_2_)). I_1_ represents intensity of array studied and I_2_ represents intensity of pseudo array containing median of intensities of all arrays. Hoeffding's statistic D_a_ was calculated to detect outlier. The top panel of figure shows 4 arrays with the highest values of D_a_ and the bottom panel show 4 arrays with the lowest values of D_a_

**Additional file 6: Table S2.** List of the primers used for qRT-PCR in this study.

**Additional file 7: Table S3.** Dataset description: This table shows the description of datasets used in this study.

**Additional file 8: Figure S5.** Schematic flowchart for ROC analyses: This schematic flowchart shows the sequential methods used in ROC analysis for the selected coding genes.

**Additional file 9: Figure S6.** Differential expression in coding genes: Volcano plots, where the differentially expressed coding genes with adjusted p-value < 0.05 and (-2) > fold change > [2] are shown for each of the three comparison (A) Chronic Pancreatitis vs. Normal Tissue (CP vs. N) (B) Pancreatic cancer vs. Normal tissue (PC vs. N) (C) Pancreatic cancer vs. Chronic Pancreatitis (PC vs. CP). Heat maps, where the expression of coding genes are shown in cases and control for each of the three comparison (E) Chronic Pancreatitis vs. Normal Tissue (CP vs. N) (E) Pancreatic cancer vs. Normal tissue (PC vs. N) (F) Pancreatic cancer vs. Chronic Pancreatitis (PC vs. CP)

**Additional file 10: Figure S7.** Differential expression in noncoding genes: Volcano plots, where the differentially expressed noncoding genes with adjusted p-value < 0.05 and (-2) > fold change > [2] are shown for each of the three comparison (A) Chronic Pancreatitis vs. Normal Tissue (CP vs. N) (B) Pancreatic cancer vs. Normal tissue (PC vs. N) (C) Pancreatic cancer vs. Chronic Pancreatitis (PC vs. CP). Heat maps, where the expression of coding genes are shown in cases and control for each of the three comparison (E) Chronic Pancreatitis vs. Normal Tissue (CP vs. N) (E) Pancreatic cancer vs. Normal tissue (PC vs. N) (F) Pancreatic cancer vs. Chronic Pancreatitis (PC vs. CP)

**Additional file 11: Table S4.** Coding DEGs-PC vs. N: This table shows the list of coding DEGs in Pancreatic cancer tissues as compared to Normal tissues

**Additional file 12: Table S5.** Coding DEGs-CP vs. N: This table shows the list of coding DEGs in Chronic Pancreatitis tissues as compared to Normal tissues

**Additional file 13: Table S6.** Coding DEGs-PC vs. CP: This table shows the list of coding DEGs in Pancreatic cancer tissues as compared to Chronic Pancreatitis tissues

**Additional file 14: Table S7.** Non-Coding DEGs-PC vs. N: This table shows the list of non-coding DEGs in Pancreatic cancer tissues as compared to Normal tissues

**Additional file 15: Table S8.** Non-Coding DEGs-CP vs. N: This table shows the list of non-coding DEGs in Chronic Pancreatitis tissues as compared to Normal tissues

**Additional file 16: Table S9.** Non-Coding DEGs-PC vs. CP: This table shows the list of non-coding DEGs in Pancreatic cancer tissues as compared to Chronic Pancreatitis tissues

**Additional file 17: Figure S8.** Selection of top malignancy specific genes: A schematic flow chart showing the selection of top malignancy specific genes from all the identified malignancy specific genes

**Additional file 18: Table S10.** Functional Annotation of genes: This table shows the functional annotation of malignancy specific genes.

**Additional file 19: Figure S9.** Correlation of genes: This figure shows correlation plot for the selected genes in the merged dataset.

## Data Availability

The raw and processed data generated during the current study has been submitted to GEO (GSE143754).

## Abbreviations

PC: Pancreatic cancer
CP: Chronic pancreatitis
TCP: Tropical calcific pancreatitis
CT: Computed tomography
MRI: Magnetic resonance imaging
F-FDG-PET: Fluorodeoxyglucose (18F)-positron emission tomography
PLR: Platelet-lymphocyte ratio
CA 19-9: Carbohydrate antigen 19-9
DEGs: Differently regulated genes
RT-PCR: Reverse transcriptase PCR
TCGA: The Cancer Genome Atlas
ROC: Receiver operating characteristics
GEO: Gene Expression Omnibus
KEGG: Kyoto Encyclopedia of Genes and Genomes
GO: Gene ontology
GAD: Genetic association database
DAVID: Database for annotation, visualization and integrated discovery
GEPIA: Gene expression profiling integrative analysis
PMM1: Phosphomannomutase 1
PAAD: Pancreatic adenocarcinoma
AUROC: Area under ROC
LASSO: Least absolute shrinkage and selection operator
KRAS: Kirsten RAS
PDAC: Pancreatic Ductal Adeno Carcinoma
N: Normal
DLGAP5: DLG associated protein 5
FOXM1: Forkhead box M1
KIF4A: Kinesin Family Member 4A
TPX2: Targeting protein for Xklp2
CDCA7: Cell division cycle associated 7
OSBPL3: Oxysterol binding protein like 3
SAMD12-AS1: SAMD12 Antisense RNA 1
MCTS2P: Malignant T cell amplified sequence 2-pseudogene
SNORD116-1: Small nucleolar RNA, C/D Box 116-1
SNORD115-15: Small nucleolar RNA, C/D Box 115-15
