# Supplementary material for: Transcriptome analysis identifies putative multi-gene signature distinguishing benign and malignant pancreatic head mass
Source: J Transl Med. 2020 Nov 7;18:420. doi: 10.1186/s12967-020-02597-1 (PMC7648960; doi:10.1186/s12967-020-02597-1)
Supplement: Supplementary file 1 — Additional file 1: Table S1. Patient characteristics: This table shows the patients characteristics for the samples used in this study [file 12967_2020_2597_MOESM1_ESM.doc]

**PATIENT CHARACTERICTS**

| Sample ID | Tumour Differentiation | Group |
| --- | --- | --- |
| R17_N | -- | N |
| R2_N | -- | N |
| R2_T | Moderately differentiated adenocarcinoma | PC |
| S108_T | Well differentiated adenocarcinoma | PC |
| S113_T | Well differentiated adenocarcinoma | PC |
| S127_N | -- | N |
| S128_N | -- | N |
| S128_T | Well differentiated adenocarcinoma | PC |
| S130_N | -- | N |
| S25_T | Well differentiated adenocarcinoma | PC |
| S27_CP | -- | CP |
| S31_CP | -- | CP |
| S35_IG | Adenocarcinoma | PC |
| S37_CP | -- | CP |
| S40_IG | Well differentiated adenocarcinoma | PC |
| S42_IG | Moderately differentiated adenocarcinoma | PC |
| S43_IG | Well differentiated adenocarcinoma | PC |
| S59_CP | -- | CP |
| S61_N | -- | N |
| S61_T | Moderately differentiated adenocarcinoma | PC |
| S71_N | -- | N |
| S71_T | Pancreatic adenocarcinoma | PC |
| S81_N | -- | N |
| S85_N | -- | N |
| S86_CP | -- | CP |
| S92_CP | -- | CP |
|  |  |  |
| S-107 | -- | CP |
| S-129 | -- | CP |
| S-142 | -- | CP |
| C-002 | -- | CP |
| S-109 | -- | CP |
| S-151 | -- | CP |
| S-114 | -- | CP |
| S-101 | -- | CP |
| S-112 | -- | CP |
| S-80 | -- | CP |
| S-178T | Moderately differentiated adenocarcinoma | PC |
| S-166T | Well Differentiated adenocarcinoma | PC |
| S-81T | Well Differentiated adenocarcinoma | PC |
| S-93T | Well Differentiated adenocarcinoma | PC |
| S-133T | Well differentiated adenocarcinoma | PC |
| S-167T | Moderately Differentiated adenocarcinoma | PC |
| S-28T | Moderately Differentiated adenocarcinoma | PC |
| CN-2T | Well differentiated adenocarcinoma | PC |
| CN-3T | Moderately differentiated adenocarcinoma | PC |
| S-146T | Moderately differentiated adenocarcinoma | PC |
