# Supplementary figures and images for: Transcriptome analysis identifies putative multi-gene signature distinguishing benign and malignant pancreatic head mass
Source: J Transl Med. 2020 Nov 7;18:420. doi: 10.1186/s12967-020-02597-1 (PMC7648960; doi:10.1186/s12967-020-02597-1)

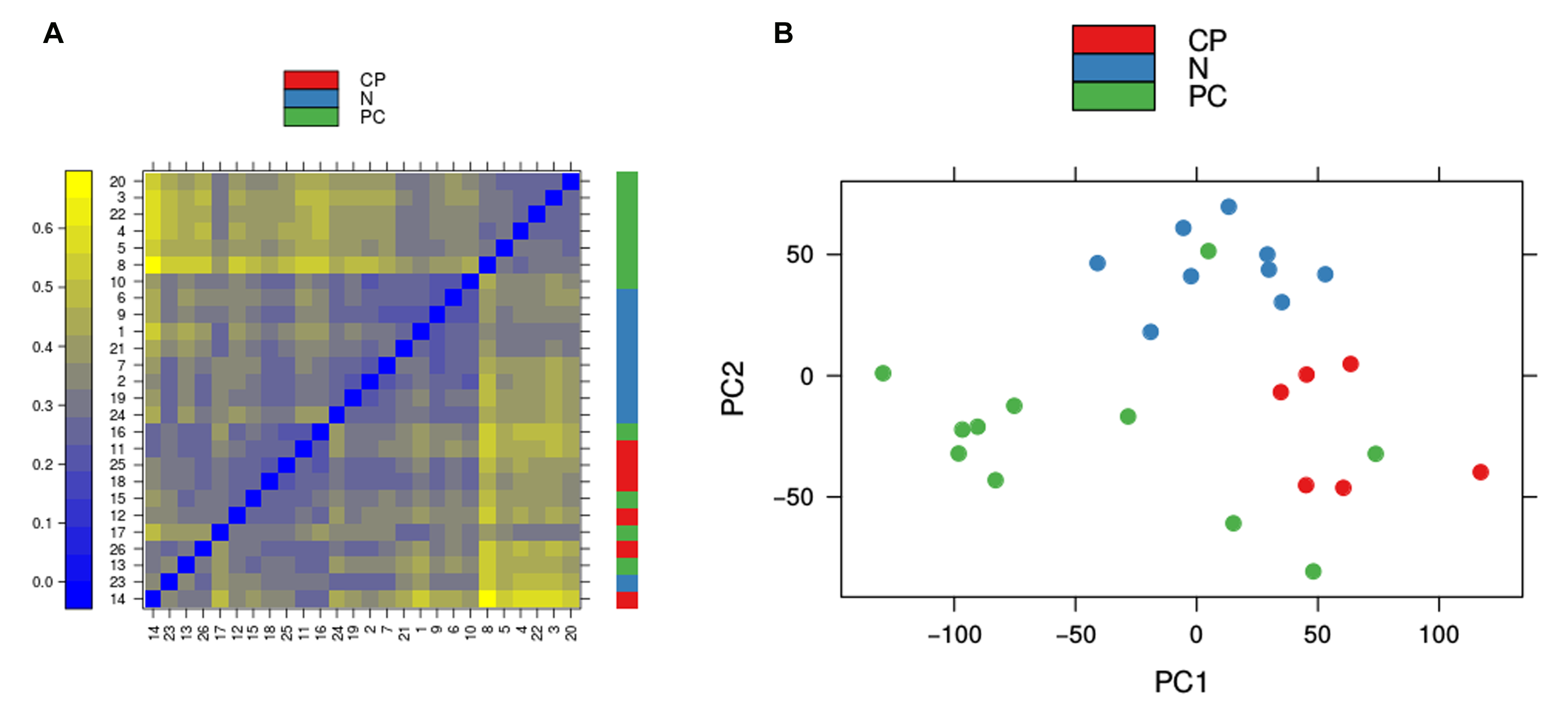

Supplement: Supplementary file 2 — Additional file 2: Figure S1. Array quality metrics-Between array comparisons: This figure shows between-array comparisons. (A) shows the distance between arrays and (B) shows Principal Component analysis (PCA). [file 12967_2020_2597_MOESM2_ESM.tif]

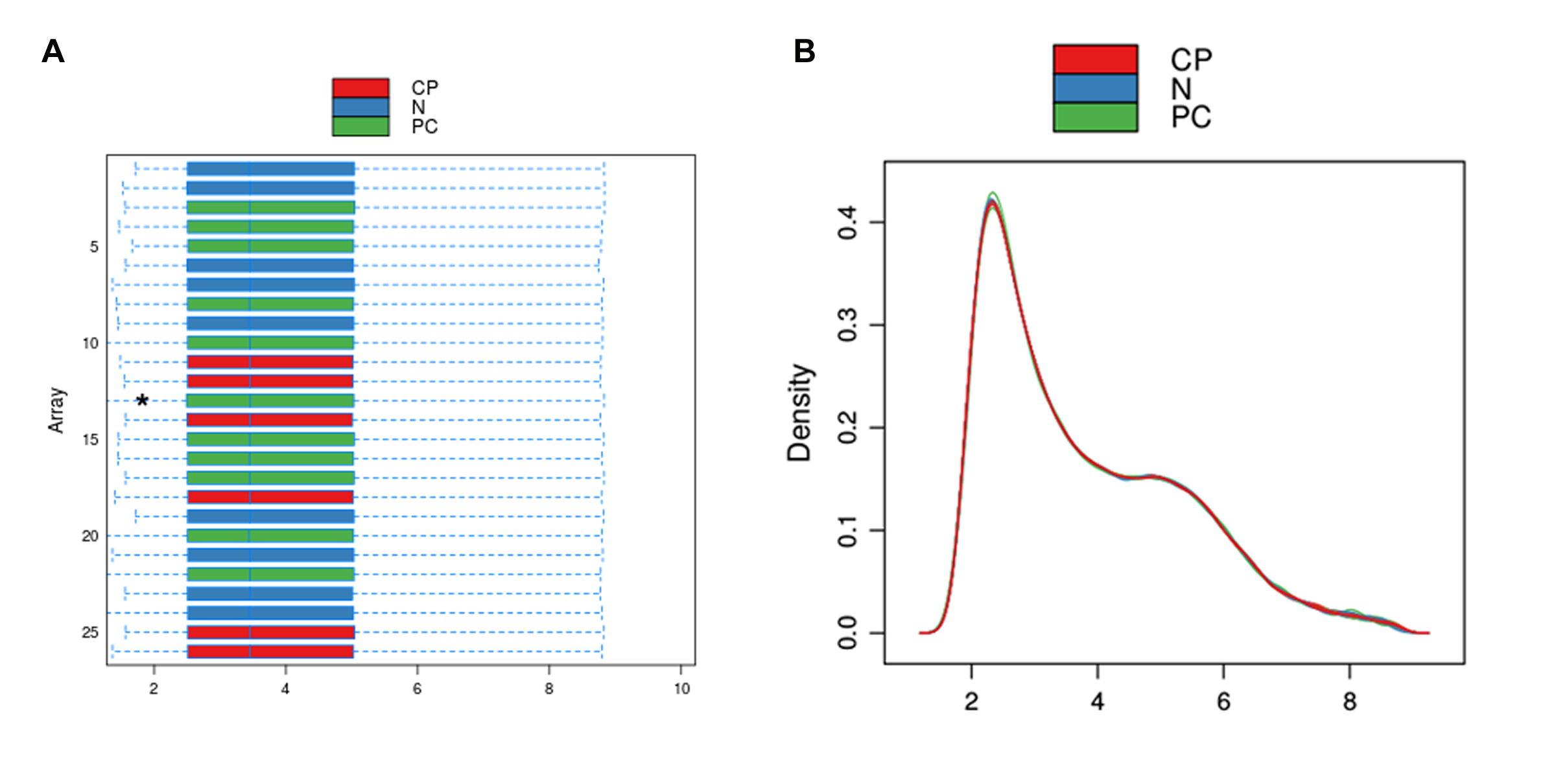

Supplement: Supplementary file 3 — Additional file 3: Figure S2. Array quality metrics-Array intensity distributions: This figure shows the array intensities in (A) boxplots and (B) density plots [file 12967_2020_2597_MOESM3_ESM.tif]

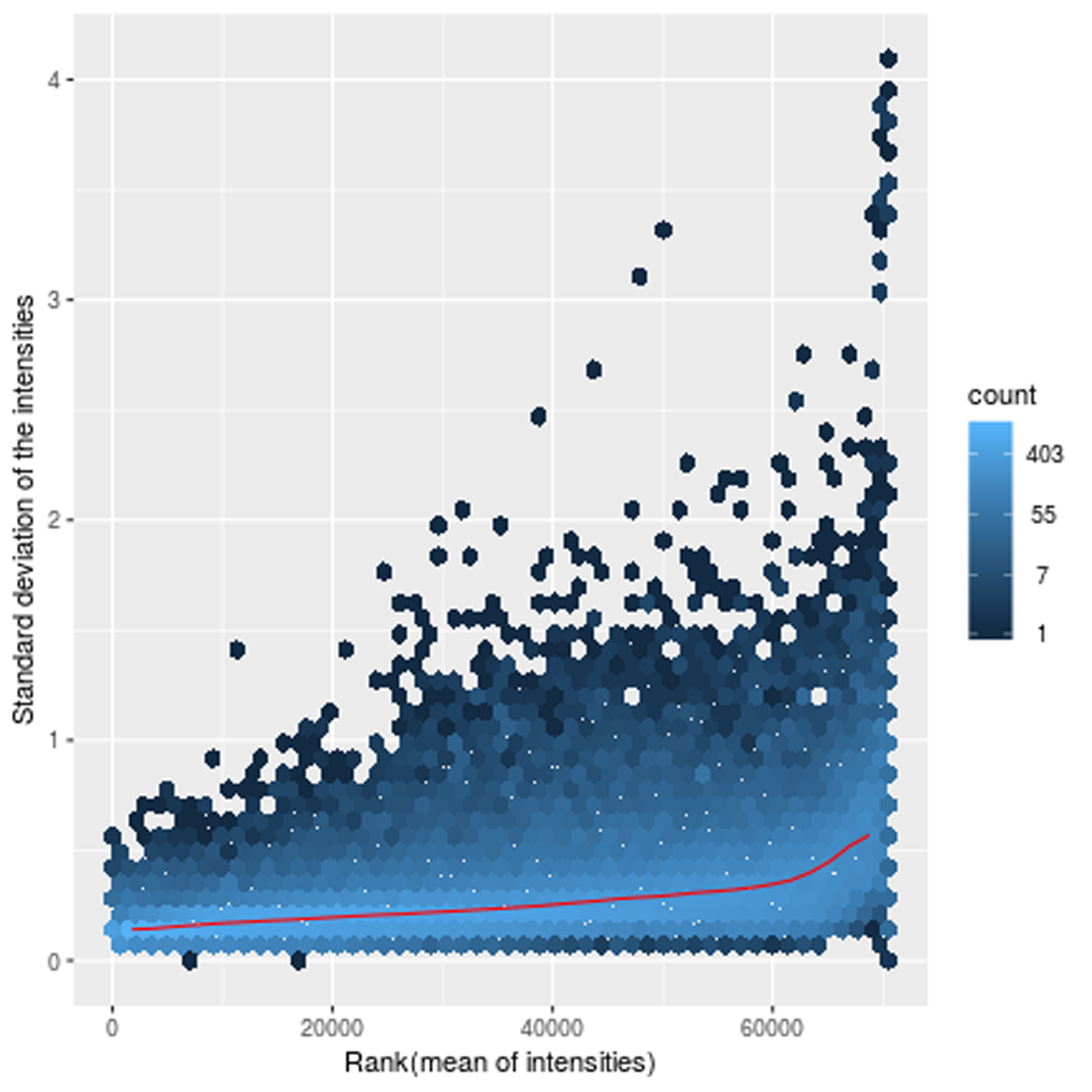

Supplement: Supplementary file 4 — Additional file 4: Figure S3. Array quality metrics-Variance Mean dependence: This figure shows the Variance Mean dependence of the arrays, where the red line connect the medians of each probe in the arrays. [file 12967_2020_2597_MOESM4_ESM.tif]

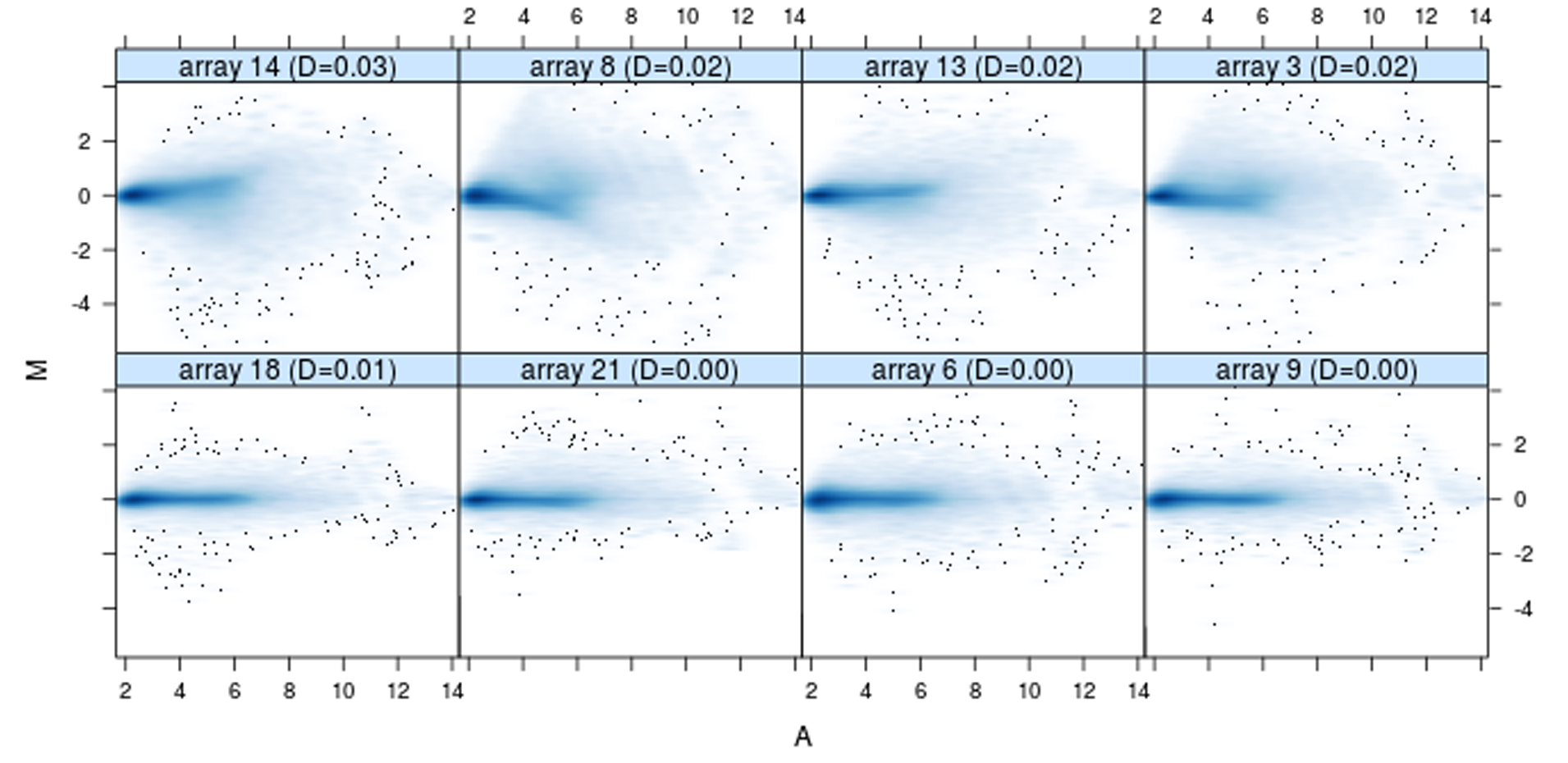

Supplement: Supplementary file 5 — Additional file 5: Figure S4. Array quality metrics-Individual array quality: This figure shows the MA plots where, M = log 2 (I1)- log 2 (I2) and A = 1/2 (log 2 (I1) + log 2 (I2)). I1 represents intensity of array studied and I2 represents intensity of pseudo array containing median of intensities of all arrays. Hoeffding's statistic Da was calculated to detect outlier. The top panel of figure shows 4 arrays with the highest values of Da and the bottom panel show 4 arrays with the lowest values of Da [file 12967_2020_2597_MOESM5_ESM.tif]

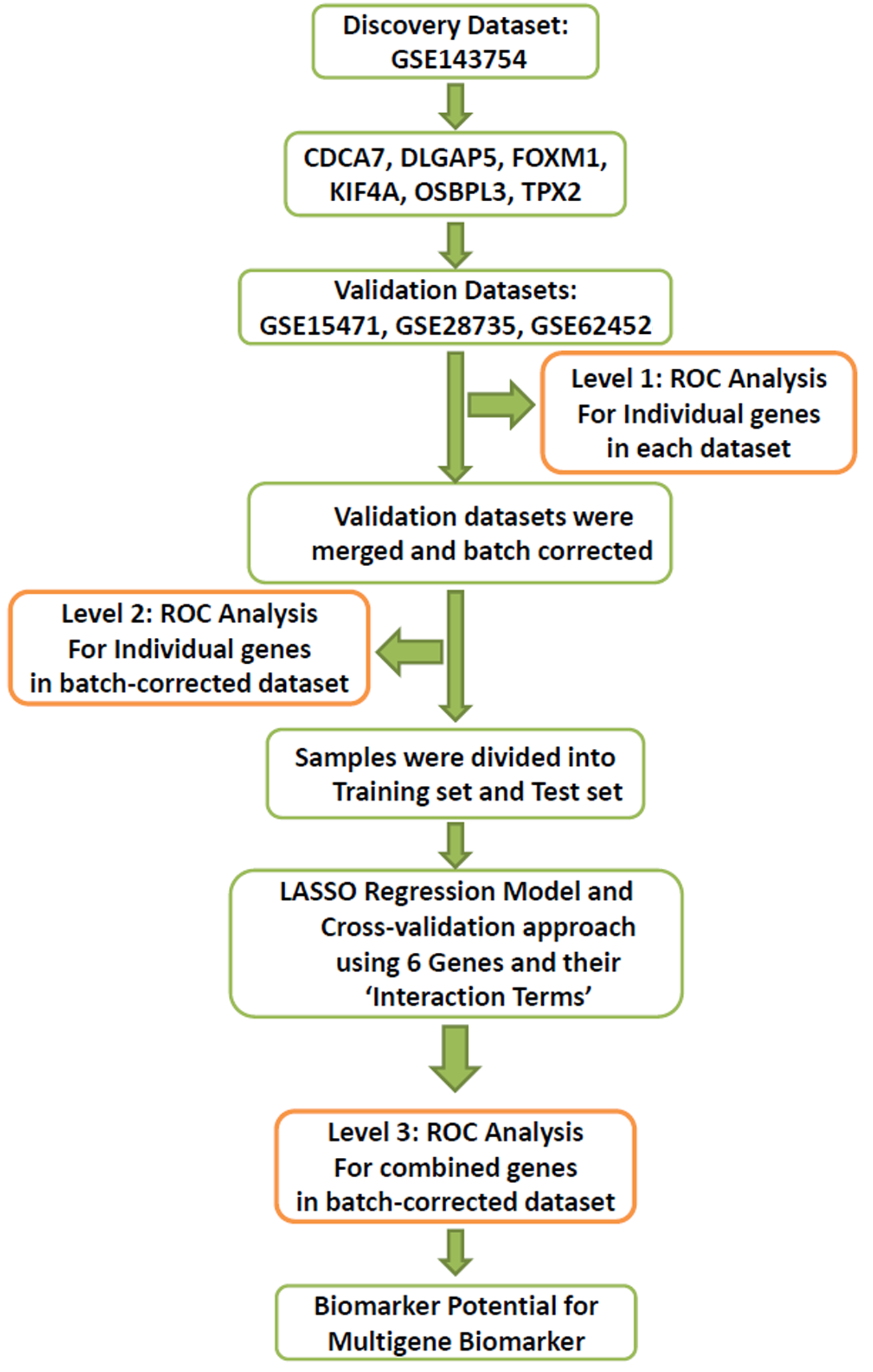

Supplement: Supplementary file 8 — Additional file 8: Figure S5. Schematic flowchart for ROC analyses: This schematic flowchart shows the sequential methods used in ROC analysis for the selected coding genes. [file 12967_2020_2597_MOESM8_ESM.tif]

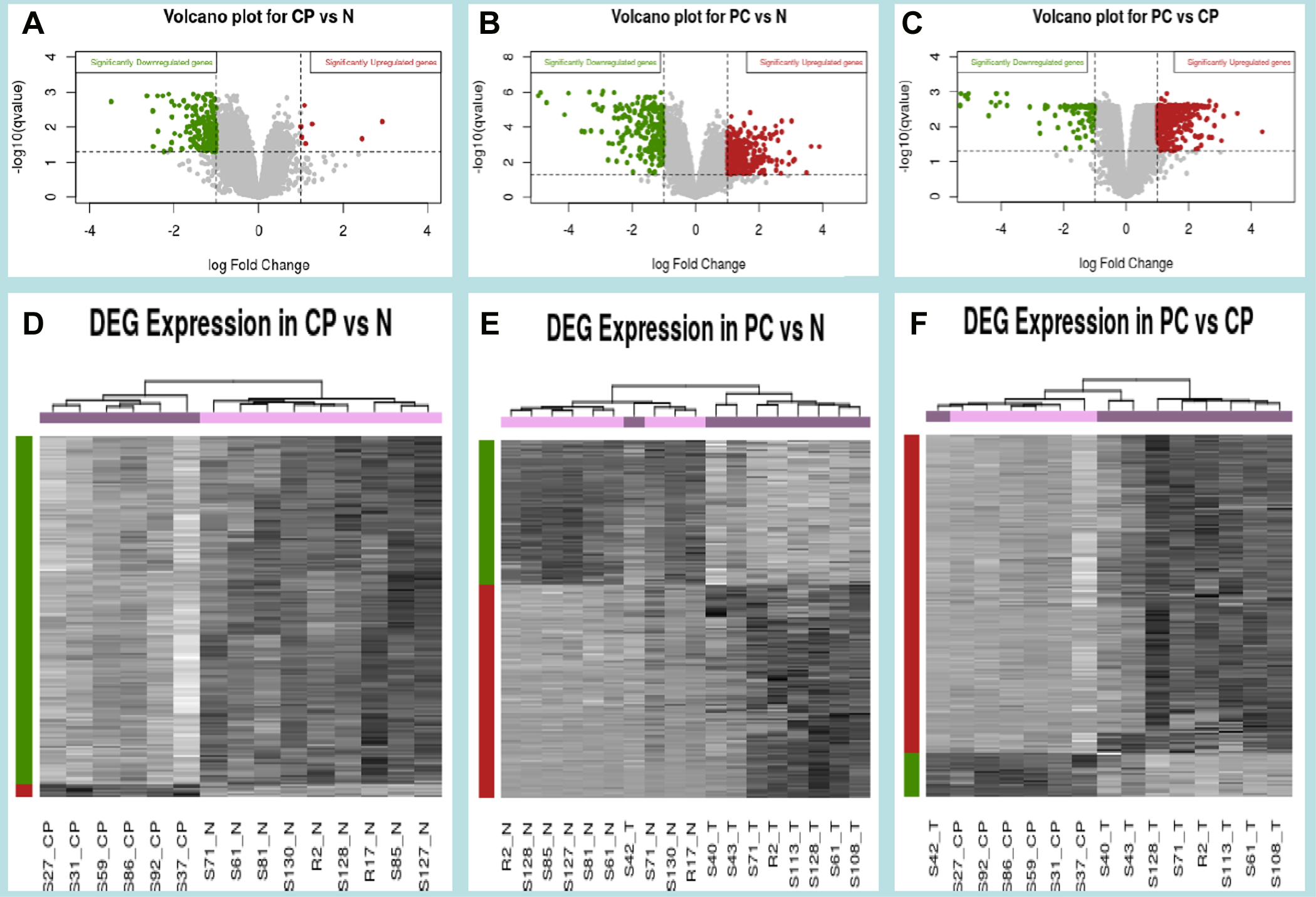

Supplement: Supplementary file 9 — Additional file 9: Figure S6. Differential expression in coding genes: Volcano plots, where the differentially expressed coding genes with adjusted p-value < 0.05 and (-2) > fold change > [2] are shown for each of the three comparison (A) Chronic Pancreatitis vs. Normal Tissue (CP vs. N) (B) Pancreatic cancer vs. Normal tissue (PC vs. N) (C) Pancreatic cancer vs. Chronic Pancreatitis (PC vs. CP). Heat maps, where the expression of coding genes are shown in cases and control for each of the three comparison (E) Chronic Pancreatitis vs. Normal Tissue (CP vs. N) (E) Pancreatic cancer vs. Normal tissue (PC vs. N) (F) Pancreatic cancer vs. Chronic Pancreatitis (PC vs. CP) [file 12967_2020_2597_MOESM9_ESM.tif]

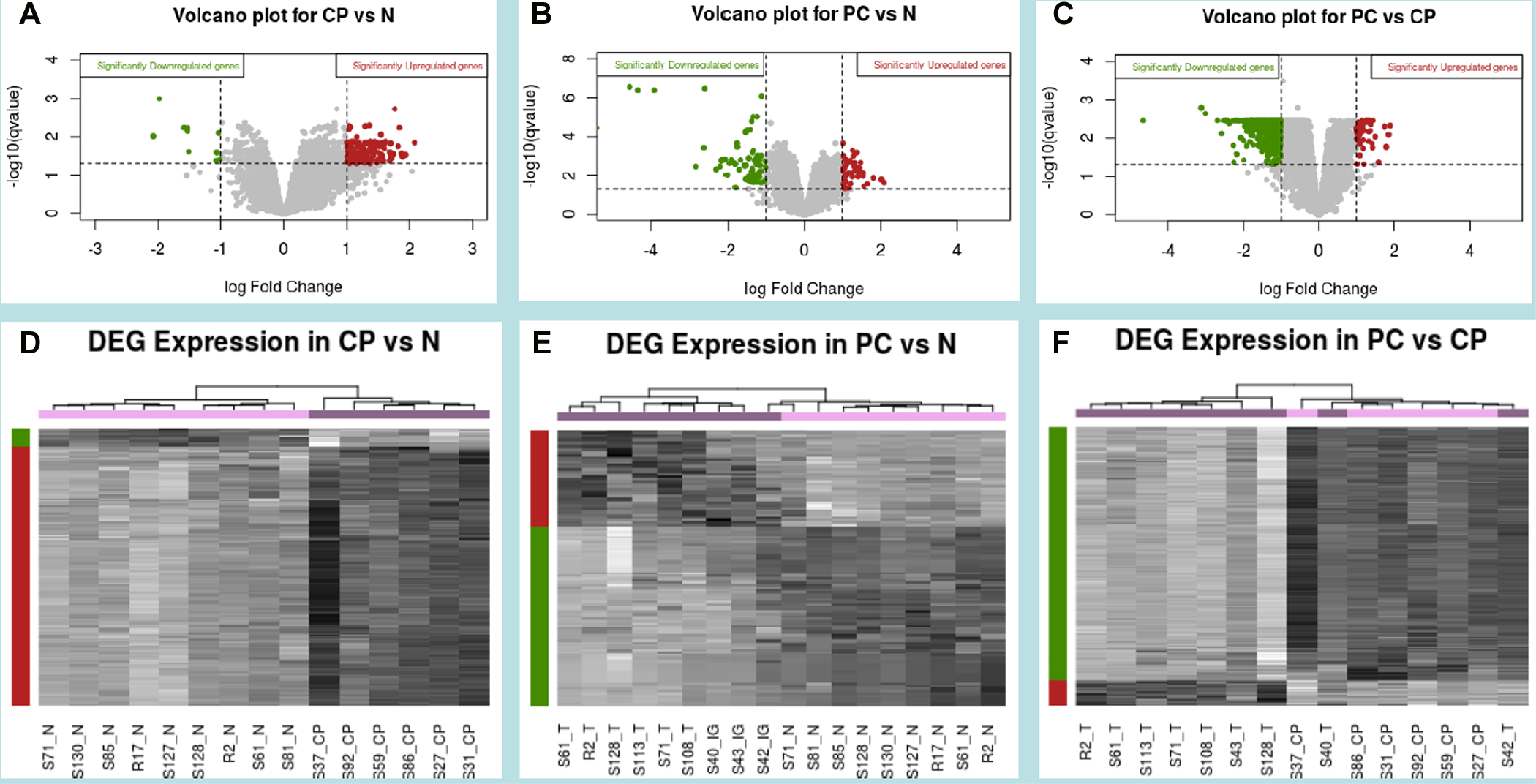

Supplement: Supplementary file 10 — Additional file 10: Figure S7. Differential expression in noncoding genes: Volcano plots, where the differentially expressed noncoding genes with adjusted p-value < 0.05 and (-2) > fold change > [2] are shown for each of the three comparison (A) Chronic Pancreatitis vs. Normal Tissue (CP vs. N) (B) Pancreatic cancer vs. Normal tissue (PC vs. N) (C) Pancreatic cancer vs. Chronic Pancreatitis (PC vs. CP). Heat maps, where the expression of coding genes are shown in cases and control for each of the three comparison (E) Chronic Pancreatitis vs. Normal Tissue (CP vs. N) (E) Pancreatic cancer vs. Normal tissue (PC vs. N) (F) Pancreatic cancer vs. Chronic Pancreatitis (PC vs. CP) [file 12967_2020_2597_MOESM10_ESM.tif]

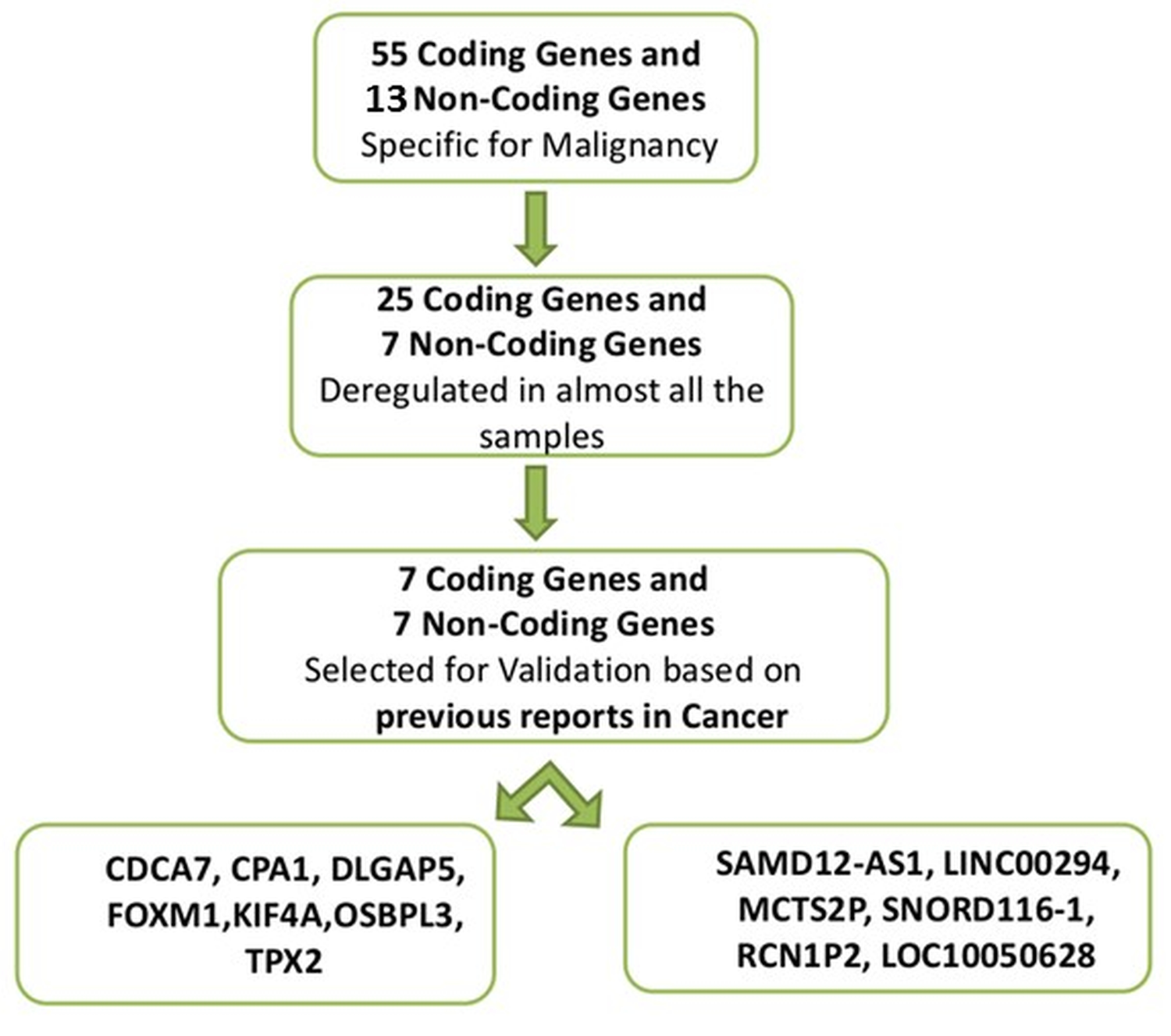

Supplement: Supplementary file 17 — Additional file 17: Figure S8. Selection of top malignancy specific genes: A schematic flow chart showing the selection of top malignancy specific genes from all the identified malignancy specific genes [file 12967_2020_2597_MOESM17_ESM.tif]

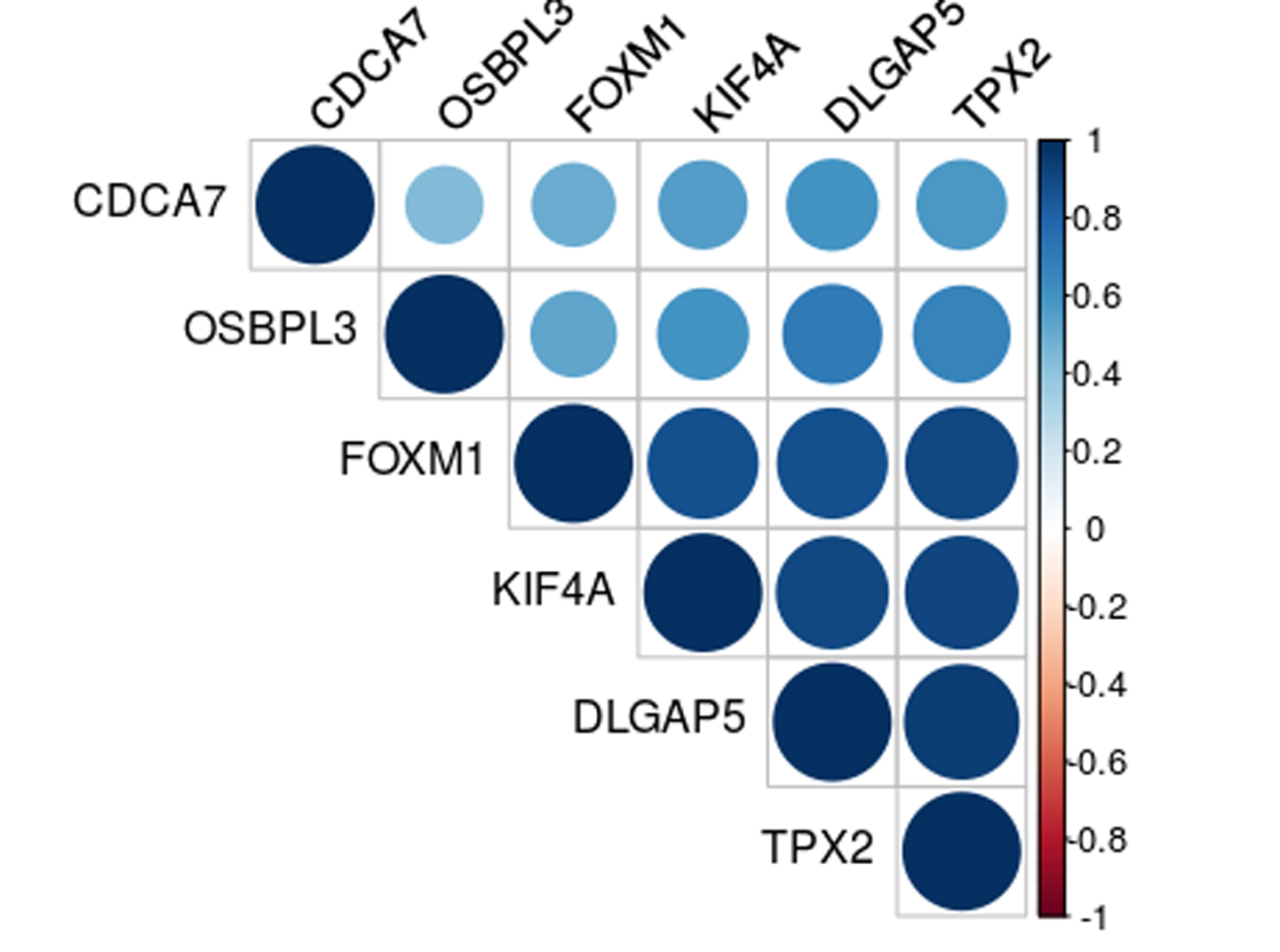

Supplement: Supplementary file 19 — Additional file 19: Figure S9. Correlation of genes: This figure shows correlation plot for the selected genes in the merged dataset. [file 12967_2020_2597_MOESM19_ESM.tif]
