# Supplementary material for: Transcriptome analysis identifies putative multi-gene signature distinguishing benign and malignant pancreatic head mass
Source: J Transl Med. 2020 Nov 7;18:420. doi: 10.1186/s12967-020-02597-1 (PMC7648960; doi:10.1186/s12967-020-02597-1)
Supplement: Supplementary file 6 — Additional file 6: Table S2. List of the primers used for qRT-PCR in this study. [file 12967_2020_2597_MOESM6_ESM.doc]

| **Gene** | **Primer** |
| --- | --- |
| PMM1_Fwd | 5’-CAGAAGCTACGAAGTAGAGTGCAGA-3’ |
| PMM1_Rev | 5’-CCGTTCTCGGCAAACACATA-3’ |
| DLGAP5_Fwd | 5’-CGACCTGGTCCAAGACAAAC-3’ |
| DLGAP5_Rev | 5’-GCTGCTTGAGTAGCTGATCG-3’ |
| FOXM1_Fwd | 5’-GCGACAGGTTAAGGTTGAG-3’ |
| FOXM1_Rev | 5’-AGGTTGTGGCGGATGGAGT-3’ |
| KIF4A_Fwd | 5’-TCAAGCAGAAACTGACCCTC-3’ |
| KIF4A_Rev | 5’-CGTTCAACAGTGCCCAAG-3’ |
| OSBPL3_Fwd | 5’-CAAACTGGACCATCCTGTCTTATG-3’ |
| OSBPL3_Rev | 5’-AAGCTAAGCACAAGTGATCATCCTAGA-3’ |
| CDCA7_Fwd | 5’-TGGAAGAAATTACAGAGGAGG-3’ |
| CDCA7_Rev | 5’-ATGACAAGTAGAGCCCAGTG-3’ |
| TPX2_Fwd | 5’-ACCTTGCCCTACTAAGATT-3’ |
| TPX2_Rev | 5’-AATGTGGCACAGGTTGAGC-3’ |
| SAMD12-AS1_Fwd | 5’-CGTCTCTCCAAAGCAACTGAA-3’ |
| SAMD12-AS1_Rev | 5’-CTTGAACTCCAGCAACTCTAGTC-3’ |
| MCTS2P_Fwd | 5’-GGAGCCTTGCCAATTCCGTTT-3’ |
| MCTS2P_Rev | 5’-ATTGCTCTACCAGTTGGCTCT-3’ |
| LOC100506281_Fwd | 5’-TTCTGCCCTTGTGGGAGTTG-3’ |
| LOC100506281_Rev | 5’-CAGCCTGCAGTTCATTTCCG-3’ |
| SNORD116-1_Fwd | 5’-TGGATCGATGATGAGTCCCC-3’ |
| SNORD116-1_Rev | 5’-TGGACCTCAGTTCCGATGAG-3’ |
| SNORD115-15_Fwd | 5’-GGGTCGATGATGAGAACCTT-3’ |
| SNORD115-15_Rev | 5’-GGGCCTCAGCGTAATCCTAT-3’ |
