# Supplementary material for: Transcriptome analysis identifies putative multi-gene signature distinguishing benign and malignant pancreatic head mass
Source: J Transl Med. 2020 Nov 7;18:420. doi: 10.1186/s12967-020-02597-1 (PMC7648960; doi:10.1186/s12967-020-02597-1)
Supplement: Supplementary file 7 — Additional file 7: Table S3. Dataset description: This table shows the description of datasets used in this study. [file 12967_2020_2597_MOESM7_ESM.doc]

**DATASET DESCRIPTION**

| **Datasets** | **Sample size** | **Description** |
| --- | --- | --- |
| GSE15471 | 39PC,39N | Whole-Tissue Gene Expression Study of Pancreatic Ductal Adenocarcinoma |
| GSE28735 | 45PC,45N | Microarray gene-expression profiles of 45 matching pairs of pancreatic tumour and adjacent non-tumour tissues from 45 patients with pancreatic ductal adenocarcinoma |
| GSE62452 | 69PC,61N | Microarray gene-expression profiles of 69 pancreatic tumours and 61 adjacent non-tumour tissue from patients with pancreatic ductal adenocarcinoma |
